# Prediction of the Ki-67 expression level in head and neck squamous cell carcinoma with machine learning-based multiparametric MRI radiomics: a multicenter study

**DOI:** 10.1186/s12885-024-12026-x

**Published:** 2024-04-05

**Authors:** Weiyue Chen, Guihan Lin, Yongjun Chen, Feng Cheng, Xia Li, Jiayi Ding, Yi Zhong, Chunli Kong, Minjiang Chen, Shuiwei Xia, Chenying Lu, Jiansong Ji

**Affiliations:** 1 Zhejiang Key Laboratory of Imaging and Interventional Medicine, The Fifth Affiliated Hospital of Wenzhou Medical University, Lishui, 323000 China; 2 Department of Radiology, The Sixth Affiliated Hospital of Wenzhou Medical University, Lishui, 323000 China; 3 Clinical College of The Affiliated Central Hospital, School of Medicine, Lishui University, Lishui, 323000 China; 4 Department of Head and Neck Surgery, The Fifth Affiliated Hospital of Wenzhou Medical University, Lishui, 323000 China

**Keywords:** Head and neck squamous cell carcinoma, Magnetic resonance imaging, Machine learning, Radiomics

## Abstract

**Background:**

This study aimed to develop and validate a machine learning (ML)-based fusion model to preoperatively predict Ki-67 expression levels in patients with head and neck squamous cell carcinoma (HNSCC) using multiparametric magnetic resonance imaging (MRI).

**Methods:**

A total of 351 patients with pathologically proven HNSCC from two medical centers were retrospectively enrolled in the study and divided into training (*n* = 196), internal validation (*n* = 84), and external validation (*n* = 71) cohorts. Radiomics features were extracted from T2-weighted images and contrast-enhanced T1-weighted images and screened. Seven ML classifiers, including k-nearest neighbors (KNN), support vector machine (SVM), logistic regression (LR), random forest (RF), linear discriminant analysis (LDA), naive Bayes (NB), and eXtreme Gradient Boosting (XGBoost) were trained. The best classifier was used to calculate radiomics (Rad)-scores and combine clinical factors to construct a fusion model. Performance was evaluated based on calibration, discrimination, reclassification, and clinical utility.

**Results:**

Thirteen features combining multiparametric MRI were finally selected. The SVM classifier showed the best performance, with the highest average area under the curve (AUC) of 0.851 in the validation cohorts. The fusion model incorporating SVM-based Rad-scores with clinical T stage and MR-reported lymph node status achieved encouraging predictive performance in the training (AUC = 0.916), internal validation (AUC = 0.903), and external validation (AUC = 0.885) cohorts. Furthermore, the fusion model showed better clinical benefit and higher classification accuracy than the clinical model.

**Conclusions:**

The ML-based fusion model based on multiparametric MRI exhibited promise for predicting Ki-67 expression levels in HNSCC patients, which might be helpful for prognosis evaluation and clinical decision-making.

**Supplementary Information:**

The online version contains supplementary material available at 10.1186/s12885-024-12026-x.

## Background

Head and neck squamous cell carcinoma (HNSCC), which arises from the mucosal epithelium of the oral cavity, larynx, and pharynx, is one of the most typical malignant tumors of the head and neck [1]. Although multimodal treatment strategies have been established in recent years, the prognosis for this highly malignant disease is still poor, and 5-year survival rates are unsatisfactory [2]. The current approach used to assess the prognosis of HNSCC patients mainly depends on the Tumor Node Metastasis (TNM) staging system. Nevertheless, due to variability in pathological features and tumor biology, even patients with the same TNM staging may have completely different survival outcomes and treatment results [3]. Therefore, it is crucial to identify a reliable prognostic indicator for HNSCC.

Ki-67 is a nuclear antigen related to cell proliferation and correlated positively with cancer aggressiveness [4]. Some studies confirmed that a high Ki-67 expression level was closely associated with the aggressive behavior and poor prognosis of HNSCC [5, 6]. In addition, prior studies demonstrated that tumors with a higher Ki-67 index were more sensitive to radiation and responded significantly better to radiation therapy [7–9]. Consequently, accurately determining the preoperative Ki-67 expression level is essential for evaluating the prognosis of HNSCC patients and clinical decision-making. In clinical practice, the Ki-67 expression level is mainly determined based on immunohistochemistry (IHC) using surgery- or biopsy-derived pathological tissues, which is invasive and time-consuming and does not enable real-time assessment. Moreover, due to the high heterogeneity of tumor tissues, local tissues obtained through biopsy alone may not accurately reflect the whole tumor [10, 11]. Therefore, an accurate and noninvasive tool is required to preoperatively assess Ki-67 expression levels in patients with HNSCC.

Magnetic resonance imaging (MRI) and computed tomography (CT) are widely used imaging modalities in the diagnosis, staging, and treatment follow-up of HNSCC. Compared with CT, MRI is considered to have substantial advantages in demonstrating the extent of tumor invasion and visualizing soft tissue [12]. However, conventional MRI results are mostly subjective and qualitative, which may lead to a lack of consistency and reproducibility between different institutions and physicians. Some quantitative parameters of functional MRI have been demonstrated to be associated with the Ki-67 expression level in HNSCC patients [13–15]. Nevertheless, these measurements are likely to be taken outside the biopsied area and may not fully reflect tumor heterogeneity. In addition, functional MRI examinations require additional scan sequences, resulting in increased costs and scan times for patients. Radiomics can extract deep quantitative features from medical images that cannot be recognized by the naked eye. By analyzing the correlation between these features and clinical, pathological, and genetic information, the overall heterogeneity and biological behavior of tumors can be unraveled [16]. In recent years, some studies have achieved a good predictive efficiency for Ki-67 in several malignant cancers, including breast cancer [17], meningioma [18], hepatocellular carcinoma [19], and sinonasal malignancy [20], using MRI-based radiomics. However, the predictive value of radiomics regarding the Ki-67 expression level in HNSCC patients remains uncertain. Moreover, multiple machine learning (ML) algorithms have not been combined with radiomics to predict Ki-67 expression in HNSCC thus far.

This study aimed to develop an ML-based radiomics model using multiparametric MRI to effectively predict the Ki-67 expression level in HNSCC patients. In addition, we constructed a fusion model based on clinical characteristics and MRI radiomics features to improve the predictive power and interpretability of the Ki-67 expression level.

## Methods

### Patient selection and clinical data

**Fig. 1:**
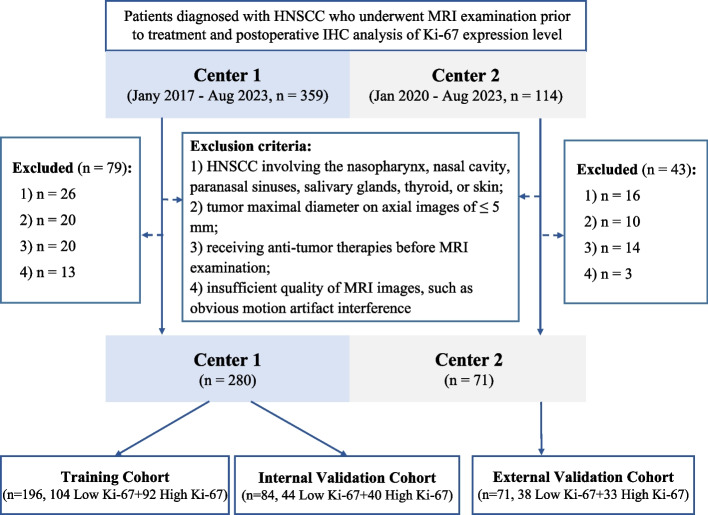
Recruitment pathway for eligible patients in this study. HNSCC, head and neck squamous cell carcinoma; IHC, immunohistochemistry; MRI, magnetic resonance imaging

This study approved by the Institutional Review Boards of the Fifth Affiliated Hospital of Wenzhou Medical University (Center 1) and the Sixth Affiliated Hospital of Wenzhou Medical University (Center 2). The requirement for informed consent from patients was waived due to the study’s retrospective nature. This study was conducted in accordance with STARD 2015 guidelines (equator-network.org). HNSCC patients with confirmed pathology were identified in Center 1 (from January 2017 to August 2023) and Center 2 (from January 2020 to August 2023). After applying the inclusion and exclusion criteria (see Appendix E1), 351 patients with HNSCC were enrolled in the study. Among them, 280 eligible patients from Center 1 were randomly divided into a training cohort (*n* = 196) and an internal validation cohort (*n* = 84) in a 7:3 ratio, while 71 patients from Center 2 were recruited as an external validation cohort. The clinical data of the enrolled patients were retrospectively collected from the medical record system and included age, sex, smoking history, tumor location, and clinical T stage. The clinical T stage of HNSCC was based on the 2017 8th edition manual of the American Joint Committee on Cancer (AJCC) [21]. The detailed patient enrollment process is shown in Fig. 1, and the sample size estimation is described in Appendix E2.

### MRI acquisition and evaluation

MR images were obtained using different 3.0-T MR scanners from two manufacturers (Center 1: Ingenia, Philips Healthcare; Center 2: Discovery 750W, GE Healthcare), both with the neck orthogonal coil. The scan sequences included T2-weighted imaging fat suppression (T2WI-FS) and contrast-enhanced T1-weighted imaging (CE-T1WI) sequences. The acquisition parameters of these protocols are summarized in Table S1. Gd-DTPA (Schering, Germany) was used as the contrast agent, injected via the arm vein with a MEDRAD high-pressure syringe at a dose of 0.2 mmol/kg and a flow rate of 2.5 ml/s. After contrast injection, 20 ml of saline was injected continuously at the same flow rate. CE-T1WI was acquired 15 s following the contrast injection.

All MRI images were reviewed in consensus by two radiologists (reader A and reader B, with 7 and 16 years of experience in head and neck MRI diagnostics, respectively) in a blinded manner (knowing the diagnosis of HNSCC but not the other clinical and pathological details). The classification criterion of MR-reported lymph node (LN) status is shown in Appendix E3.

### IHC staining of Ki-67

**Fig. 2:**
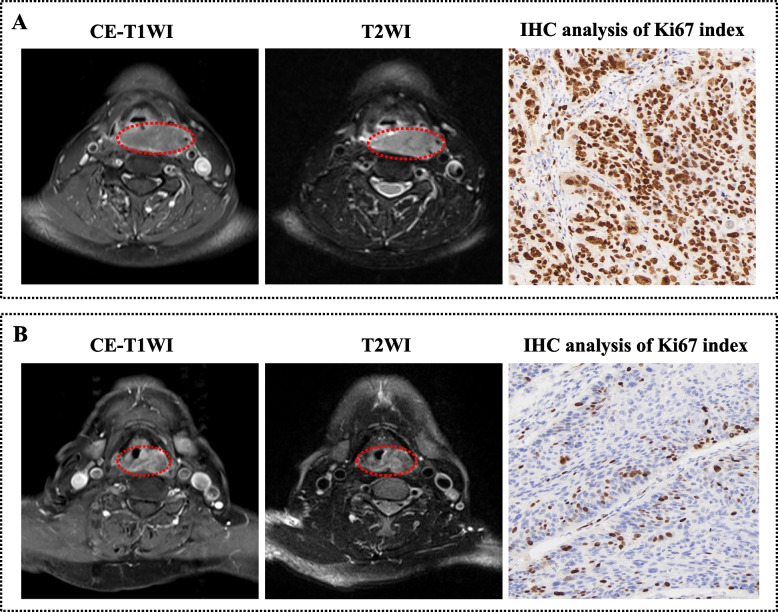
Representative MRI images of head and neck squamous cell carcinoma patients whose Ki-67 expression levels were determined as being low and high. **A** A 67-year-old male patient with a visible mass in the hypopharynx. The immunohistochemical image presented a low Ki-67 expression level (× 200; Ki-67 index = 20%). **B** A 62-year-old male patient with a visible mass in the hypopharynx. The immunohistochemical image presented a high Ki-67 expression level (× 200; Ki-67 index = 90%). MRI, magnetic resonance imaging

The expression level of Ki-67 was assessed by performing IHC staining on surgical histopathology samples. After sample fixation, embedding, drying, dewaxing, rinsing, and hydration, IHC staining was performed using a Ki-67 protein antibody (dilution 1:300). Cells were considered positive when the nuclei were dark yellow or brown. Positive cells were selected from among the five areas with the highest density of positives, following which 100 nuclei were counted at a high magnification (× 200) to determine the percentage of positive cells. According to previous studies [22–24], using 50% as the cut-off value of Ki-67 in HNSCC can effectively predict the prognosis. Thus, a Ki-67 index of < 50% was considered low expression, while that of ≥ 50% was defined as high expression. The Ki-67 analyses were retrospectively performed by two pathologists (with 5 and 10 years of experience) who were blinded to the clinical information. Representative MRI images from patients with Ki-67 expression levels identified as low and high are shown in Fig. 2.

### Tumor segmentation, feature extraction, and repeatability analysis

**Fig. 3:**
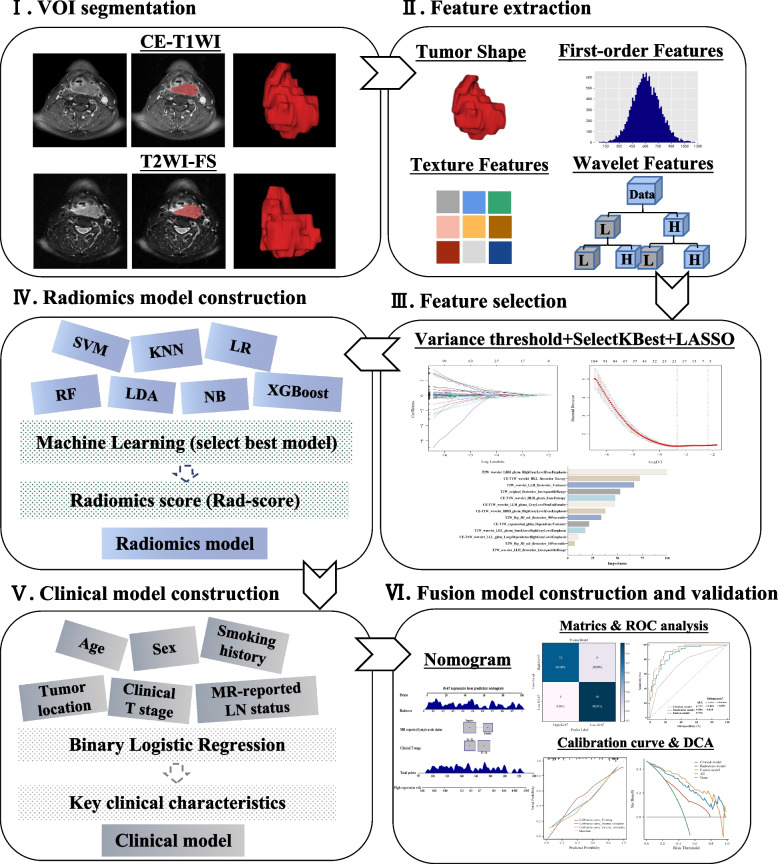
The workflow of radiomics analysis in the present study. First, VOIs were manually delineated around the entire tumor outline on each axial slice of T2WI-FS and CE-T1WI images. Second, 1688 radiomics features were extracted from each three-dimensional segmentation. Third, three steps of feature selection were applied to all extracted features. Then, seven radiomics signatures were built using seven machine learning classifiers, and the radiomics signature with the best predictive performance was used to build the radiomics model. A clinical model was constructed using logistic regression analysis. Finally, a fusion model incorporating the optimal radiomics score and key clinical characteristics was built and presented as a nomogram, which was evaluated by ROC analysis, calibration curve, and DCA. CE-T1WI, contrast-enhanced T1-weighted imaging; DCA, decision curve analysis; LASSO, least absolute shrinkage and selection operator; ROC, receiver operating characteristic; T2WI-FS, T2-weighted imaging fat suppression; VOI, volume of interest

The radiomics workflow of the present study is shown in Fig. 3. Bias field correction was performed to eliminate signal intensity variations due to magnetic field inhomogeneities before outlining the regions of interest (ROIs). The pre-processed images were uploaded to the Radcloud platform (v7.1, http://radcloud.cn/). Two radiologists (reader A and reader B) who were blinded to the final pathological results outlined ROIs along the edges of the lesion layer-by-layer on the T2WI-FS and CE-T1WI images, respectively. For each sequence image, a separate whole-tumor volume of interest (VOI) was generated by ROI superimposition.

Then, the radiomics features from each VOI were extracted using the Radcloud platform with a wide variety of engineered, hard-coded feature algorithms [25, 26]. Before feature extraction, all images were resampled to a voxel size of 1 × 1 × 1 mm^3^ using B-Spline interpolation to reduce the effect of slice thickness variations and isotropic voxels to ensure rotation invariance. Subsequently, to minimize inherent differences in pixel intensities across two different MR scanners, the gray-level intensity for all image volumes was scaled in the range of 0–255 after removing pixels with outlier values [27]. A total of 1688 radiomics features were initially extracted from each VOI for each patient image sequence. The details of the extracted features are given in Appendix E4. The intra- and inter-class correlation coefficients (ICCs) were calculated for repeatability analysis (Appendix E5).

### Feature selection, radiomics signature construction, and evaluation

To eliminate scaling differences, radiomics features were normalized using a standardized method (Appendix E6). Then, a three-step procedure involving variance threshold, SelectKBest, and least absolute shrinkage and selection operator (LASSO) regression was performed for the selection of task-specific radiomics features in the training cohort from the feature subsets of T2WI-FS and CE-T1WI sequences alone and in combination, as detailed in Appendix E7.

Thereafter, the selected features were entered into the following ML classifiers to construct radiomics signatures: k-nearest neighbors (KNN), support vector machine (SVM), logistic regression (LR), random forest (RF), linear discriminant analysis (LDA), naive Bayes (NB), and eXtreme Gradient Boosting (XGBoost). The rationales and considerations behind the choice of the seven ML classifiers in this study were detailed in the Appendix E8. The Grid Search in Python was utilized to automatically search for the optimal hyperparameter combinations for each classifier (see in the Appendix E9). Additionally, the seven classifiers were validated in the validation cohorts. The prediction performance of the radiomics signatures was evaluated using the area under the receiver operator characteristic (ROC) curve (AUC), sensitivity, specificity, and accuracy. The classifier with the highest average AUC value in the validation cohorts was chosen as the best classifier [28–30]. The best classifier was used to classify key radiomics features of HNSCC patients according to different Ki-67 expression levels, thereby calculating Radiomics (Rad)-scores, which indicate the relative risk of high Ki-67 expression in HNSCC patients, and were used to build the radiomics model. The distribution of the Rad-scores between the Ki-67 low- and high-expression groups was also analyzed to verify its diagnostic performance.

### Development and validation of the prediction models

Univariate LR analysis was performed to assess the association between clinical-radiological characteristics and Ki-67 expression level, and clinical predictors with *P* < 0.1 were included in the multivariate LR analysis to develop the clinical model. Afterward, multivariate analysis and backward stepwise regression analysis based on the Akaike Information Criterion were performed to establish the fusion model and corresponding nomogram incorporating the Rad-score and significant clinical predictors in the training cohort. During this procedure, collinearity was examined, and variables with a variance inflation factor (VIF) of greater than 10 and *P* > 0.05 were excluded [31]. The models were tested in the validation cohorts. The predictive performance of the prediction models was evaluated using ROC analysis, calibration curves, and decision curve analysis (DCA). The percentage of true positive, false positive, true negative, and false negative results was determined according to the reference standard of pathological results by ROC analysis, and the results are displayed in the form of a confusion matrix diagram. Calibration curves were plotted by bootstrapping with 1000 resamples, and DCA was performed to visualize the net benefit for clinical decisions. The net reclassification improvement (NRI) and integrated discrimination improvement (IDI) values were used to quantify the different models’ clinical usefulness and net benefit.

### Statistical analysis

All statistical analyses were completed using Python v3.7.6 and R software. The Kolmogorov–Smirnov test was performed to test the normality of continuous variables. Student’s *t*-test was applied to compare continuous variables with a normal distribution, the Mann–Whitney *U* test was used for non-normally distributed variables, and the chi-square test was performed for categorical variables. The R packages used in this study included “glmnet” (for LASSO regression), “rms” (for LR analysis and calibration curves), “rmda” (for DCA), and “PredictABEL” (for the calculation of NRI and IDI). ROC analysis was performed using MedCalc, and the DeLong test was used to compare the differences in AUC values between models. All tests were two-tailed, and *P* < 0.05 was considered statistically significant.

## Results

### Patient characteristics and clinical model construction

The clinical characteristics and MRI features of the 351 patients in the training, internal validation, and external validation cohorts are summarized in Table 1 and Table S2. Overall, the three cohorts were balanced and comparable. Significant differences in the clinical T stage and MR-reported LN status were observed between the Ki-67 low- and high-expression groups in all three cohorts (all *P* < 0.05), while differences in other characteristics were not statistically significant (all *P* > 0.05). Following univariate and multivariate regression analyses, clinical T3-T4 stage (odds ratio [OR]: 3.715, confidence interval [CI]: 1.580–8.737,* P* = 0.003) and MR-reported LN metastasis (OR: 2.836, CI: 1.195–6.729, *P* = 0.018) were confirmed as independent predictors of high Ki-67 expression and used to construct the clinical model (Table S3). No collinearity was detected since the VIFs of the predictors were 1.077 and 1.131, respectively.

**Table 1:** Comparison of clinical characteristics and MRI features between patients with Ki-67 low expression and those with high expression of HNSCC

Characteristic	Training cohort (*n* = 196)		*P* value	Internal Validation cohort (*n* = 84)		*P* value	External Validation cohort (*n* = 71)		*P* value
	Low Ki-67 expression (*n* = 104)	High Ki-67 expression (*n* = 92)		Low Ki-67 expression (*n* = 44)	High Ki-67 expression (*n* = 40)		Low Ki-67 expression (*n* = 38)	High Ki-67 expression (*n* = 33)	
Age, years			0.495			0.785			0.702
< 60	48 (46.1)	38 (41.3)		20 (45.5)	17 (42.5)		19 (50.0)	15 (45.5)	
≥ 60	56 (53.8)	54 (58.7)		24 (54.5)	23 (57.5)		19 (50.0)	18 (54.5)	
Sex			0.187			0.403			0.571
Male	85 (81.7)	68 (73.9)		33 (75.0)	33 (82.5)		29 (76.3)	27 (81.8)	
Female	19 (18.3)	24 (26.1)		11 (25.0)	7 (17.5)		9 (23.7)	6 (18.2)	
Smoking history			0.767			0.749			0.592
No	60 (57.7)	55 (59.8)		26 (59.1)	25 (62.5)		23 (60.5)	22 (66.7)	
Yes	44 (42.3)	37 (40.2)		18 (40.9)	15 (37.5)		15 (39.5)	11 (33.3)	
Tumor location			0.466			0.226			0.113
Oral cavity	42 (40.4)	33 (35.9)		20 (45.4)	13 (32.5)		18 (47.4)	10 (30.3)	
Oropharynx	11 (10.6)	17 (18.5)		7 (15.9)	4 (10.0)		6 (15.8)	3 (9.1)	
Larynx	35 (33.6)	28 (30.4)		15 (34.1)	17 (42.5)		12 (31.6)	13 (39.4)	
Hypopharynx	16 (15.4)	14 (15.2)		2 (4.5)	6 (15.0)		2 (5.3)	7 (21.2)	
Clinical T stage			< 0.001			0.004			0.006
T1-T2	63 (60.6)	24 (26.1)		28 (63.6)	13 (32.5)		24 (63.2)	10 (30.3)	
T3-T4	41 (39.4)	68 (73.9)		16 (36.4)	27 (67.5)		14 (36.8)	23 (69.7)	
MR-reported LN status			< 0.001			0.005			0.029
Negative	78 (75.0)	42 (45.7)		33 (75.0)	18 (45.0)		27 (71.1)	15 (45.5)	
Positive	26 (25.0)	50 (54.3)		11 (25.0)	22 (55.0)		11 (28.9)	18 (54.5)	

*HNSCC* Head and neck squamous cell carcinoma, *LN* Lymph node, *MRI* Magnetic resonance imaging

### Radiomics feature selection and signature construction

Intra-observer ICCs ranged from 0.858 to 0.963, while inter-observer ICCs ranged from 0.827 to 0.931. The results of feature selection in the training cohort are shown in Fig. S1. The optimal radiomics feature subsets selected from T2WI-FS and CE-T1WI for predicting high Ki-67 expression are listed in Table S4. Finally, 13 radiomics features were retained from the combined images of T2WI-FS (*n* = 7) and CE-T1WI (*n* = 6) using LASSO regression (Fig. S2), and the relative importance of the 13 selected features is shown in Fig. S2C. The correlation heatmap indicated that the selected features from the combined images were relatively independent (Fig. S3). Then, all selected features were combined to generate the radiomics signature with seven ML classifiers (KNN, SVM, LR, RF, LDA, NB, and XGBoost).

### Performance of radiomics signatures and radiomics model construction

**Fig. 4:**
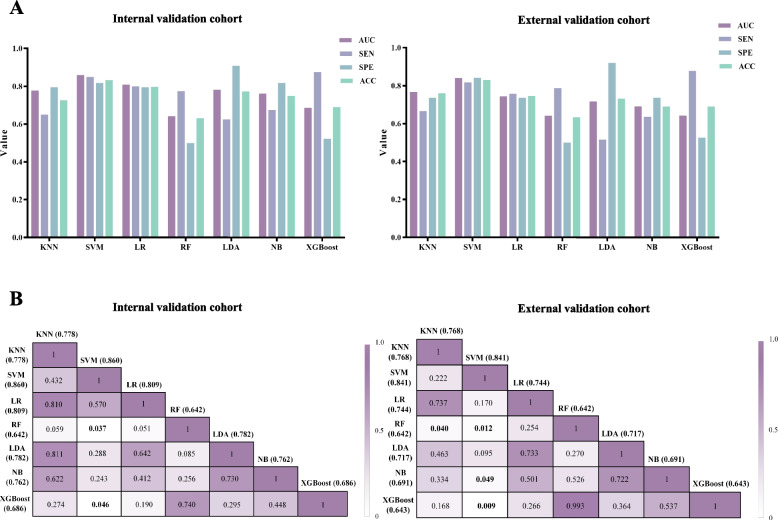
ROC analysis results **(A)** and DeLong’s tests (*P* value) of different radiomics signatures **(B)** in the internal validation cohort (left) and external validation cohort (right). ACC, accuracy; AUC, area under the curve; KNN, k-nearest neighbors; LDA, linear discriminant analysis; LR, logistic regression; NB, naive Bayes; RF, random forest; ROC, receiver operating characteristic; SEN, sensitivity; SPE, specificity; SVM, support vector machine; XGBoost, eXtreme Gradient Boosting

For the combined sequences, the predictive performances of the radiomics signatures based on six classifiers in the training, internal validation, and external validation cohorts are shown in Fig. 4 and Table 2. Among these classifiers, the accuracy of RF was 100.0% in the training cohort but 60.2% and 63.4% in the internal and external validation cohorts, respectively, which suggested the presence of overfitting. The SVM classifier achieved the highest average AUC of 0.851 and the highest average accuracy of 0.832 in the validation cohorts. Moreover, the SVM classifier exhibited better predictive performance than the other classifiers in the validation cohorts according to the ROC curves (Fig. S4) and DeLong tests (Fig. 4B). Therefore, SVM was selected as the optimal classifier to calculate Rad-scores for constructing the radiomics model.

**Table 2:** Diagnostic performance of various machine learning-based radiomics signatures

Model	Training cohort		Internal validation cohort		External validation cohort		Validation cohorts	
	AUC (95% CI)	ACC (%)	AUC (95% CI)	ACC (%)	AUC (95% CI)	ACC (%)	Average AUC	Average ACC (%)
KNN	0.829 (0.753—0.904)	77.55	0.778 (0.675—0.862)	72.62	0.768 (0.653—0.860)	76.06	0.773	74.34
SVM	0.884 (0.831—0.925)	80.61	0.860 (0.767—0.926)	83.33	0.841 (0.734—0.917)	83.10	0.851	83.22
LR	0.870 (0.815—0.914)	82.14	0.809 (0.708—0.886)	79.76	0.744 (0.627—0.840)	74.64	0.777	77.20
RF	1.000 (1.000–1.000)	100.0	0.642 (0.530—0.744)	63.10	0.642 (0.519—0.752)	63.38	0.642	63.24
LDA	0.830 (0.770—0.880)	75.00	0.782 (0.678—0.864)	77.38	0.717 (0.598—0.818)	73.24	0.749	75.31
NB	0.769 (0.704—0.826)	70.92	0.762 (0.657—0.848)	75.00	0.691 (0.571—0.796)	69.01	0.727	72.01
XGBoost	0.926 (0.880—0.959)	85.71	0.686 (0.575—0.783)	69.05	0.643 (0.520—0.753)	69.01	0.665	69.03

*AUC* Area under the curve, *ACC* Accuracy, *CI* Confidence interval, *KNN* K-nearest neighbors, *SVM* Support vector machine, *LR* Logistic regression, *RF* Random forest, *LDA* Linear discriminant analysis, *NB* Naive Bayes, *XGBoost* eXtreme Gradient Boosting

We constructed the radiomics models using the single sequence (T2WI-FS and CE-T1WI) images based on the SVM classifier. As depicted in Fig. S5, the radiomics model using the combined sequences had higher AUC values than the models with T2WI-FS and CE-T1WI in all three cohorts (all *P* < 0.05). The SVM-based Rad-scores using combined sequences showed significant differences between the Ki-67 low- and high-expression groups in all three cohorts (all *P* < 0.001, Fig. S6A-C), and the correlation between Ki-67 status, clinical features, and radiomics features is shown in Fig. S6D-F.

### Development and validation of an individualized prediction nomogram

**Fig. 5:**
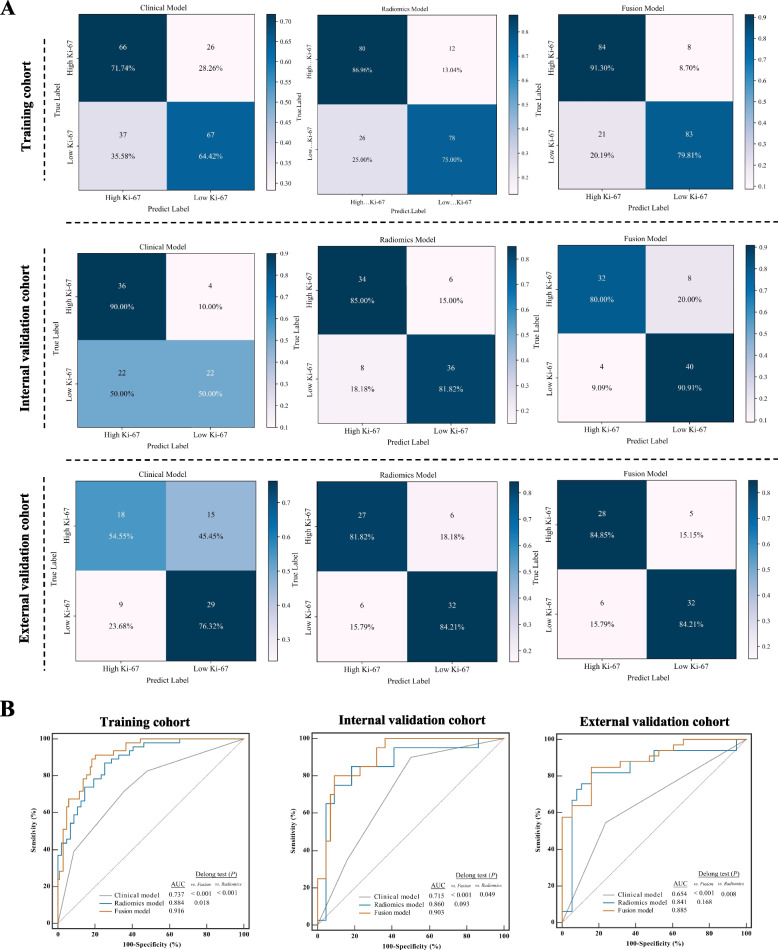
**A** Confusion matrices and (**B**) ROC curves of Ki-67 expression level classification for different models in the training, internal validation, and external validation cohorts. **A** Confusion matrices of the clinical model, radiomics model, and fusion model. The color depends on the number inside the square: the higher the number, the darker the color. **B** ROC curves of different models for predicting Ki-67 expression levels and the results of DeLong’s tests. AUC, area under the curve; ROC, receiver operating characteristic

We further integrated the SVM-based Rad-scores with significant clinical factors (clinical T stage and MR-reported LN status) to build a fusion prediction model. The detailed performance of three models in the training and validation cohorts is summarized in Table 3 and depicted in Fig. 5A by confusion matrices. The ROCs for Ki-67 status prediction according to these three models and the results of DeLong’s tests are shown in Fig. 5B. Notably, the incorporation of Rad-scores led to a significant increase in the AUC values for the clinical model in the training, internal validation, and external validation cohorts from 0.737 to 0.916 (Z = 5.702, *P* < 0.001), 0.715 to 0.903 (Z = 3.485, *P* = 0.011), and 0.654 to 0.885 (Z = 3.477, *P* < 0.001), respectively. However, no significant difference in AUCs was found between the radiomics model and fusion model in the internal and external validation cohorts (all *P* > 0.05).

**Table 3:** Predictive performance of the clinical model, SVM-based radiomics model and fusion model in the training, internal validation and external validation cohorts

Cohort	Model	AUC (95% CI)	SEN (%)	SPE (%)	ACC (%)
Training	Clinical	0.737 (0.669—0.797)	71.74	64.42	67.86
	SVM-based radiomics	0.884 (0.831—0.925)	86.96	75.00	80.61
	Fusion	0.916 (0.868—0.951)	91.30	79.81	85.20
Internal validation	Clinical	0.715 (0.606—0.808)	90.00	50.00	69.05
	SVM-based radiomics	0.860 (0.767—0.926)	85.00	81.82	83.33
	Fusion	0.903 (0.819—0.957)	80.00	90.91	85.71
External validation	Clinical	0.654 (0.532—0.763)	54.55	76.32	66.20
	SVM-based radiomics	0.841 (0.734—0.917)	81.82	84.20	83.10
	Fusion	0.885 (0.787—0.949)	84.85	84.21	84.51

*AUC* Area under the curve, *ACC* Accuracy, *CI* Confidence interval, *SEN* Sensitivity, *SPE* Specificity, *SVM* Support vector machine

**Fig. 6:**
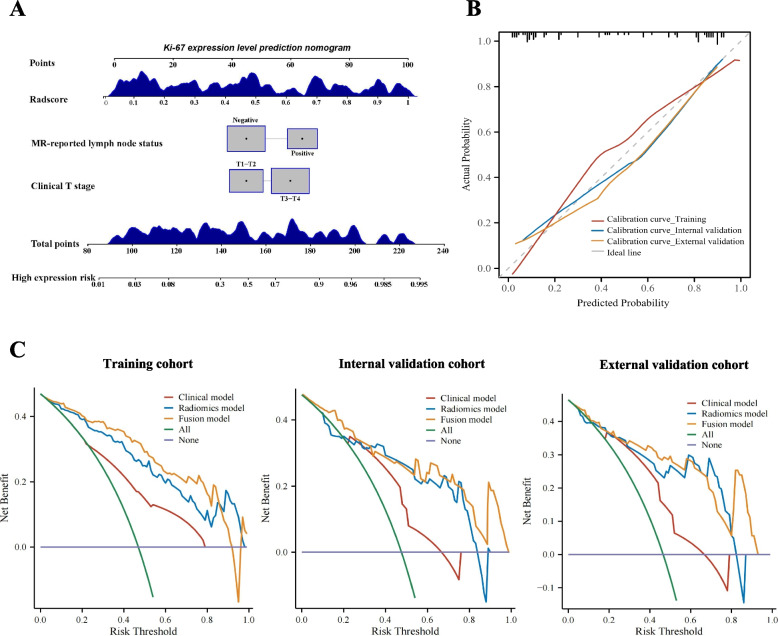
The nomogram, calibration curves, and DCA. **A** The fusion nomogram incorporating the SVM-based Rad-score and clinical characteristics (clinical T stage and MR-reported lymph node status) for predicting the probability of high Ki-67 expression (Ki-67 index ≥ 50%). **B** The calibration curves of the fusion model in the training, internal validation, and external validation cohorts. **C** The DCA results of the clinical model, radiomics model, and fusion model in the three cohorts. DCA, decision curve analysis; MR, magnetic resonance; SVM, support vector machine

Finally, we visualized the fusion model as a nomogram to individually predict the risk of high Ki-67 expression in HNSCC patients (Fig. 6A). The calibration curves showed that the predicted Ki-67 high-expression probabilities of the fusion model had excellent agreement with the actual observations (Fig. 6B). Additionally, the DCA results showed that the radiomics model and fusion model had a higher overall net benefit than the clinical model across the majority of the range of reasonable threshold probabilities in the three cohorts (Fig. 6C). Furthermore, the inclusion of Rad-scores in the fusion model yielded a total NRI of 0.477 (95% CI: 0.206–0.748, *P* < 0.05) and IDI of 0.204 (95% CI: 0.112–0.325, *P* < 0.05). Similar results were observed in the validation cohorts (Fig. S7), which showed improved prediction efficiency and classification accuracy for the Ki-67 expression outcome. However, we found no significant difference in the NRI and IDI between the radiomics model and the fusion model in the three cohorts (all *P* > 0.05).

## Discussion

High Ki-67 expression in HNSCC correlates with strong proliferative activity and tumor invasiveness [5]. Additionally, the Ki-67 index can be used as an important indicator to help identify candidates for radiotherapy [7]. Thus, the accurate preoperative assessment of Ki-67 expression is essential for prognostic evaluation and treatment planning. This is the first study to establish a fusion model using multiparametric MRI to preoperatively predict the Ki-67 expression level in HNSCC patients by incorporating SVM-based radiomics signatures and clinical features. The fusion model accurately distinguished between Ki-67 indexes of < 50% and ≥ 50% with favorable AUCs (0.916, 0.903, and 0.885, respectively) and high accuracies (85.20%, 85.71%, and 84.51%, respectively) in the training, internal validation, and external validation cohorts. The proposed fusion model had superior performance to the clinical model that included clinical T stage and MRI-reported LN, suggesting that the addition of radiomics features enhanced its diagnostic efficacy and incremental value in predicting Ki-67 expression level. Thus, this model can accurately and robustly predict high Ki67 expression in HNSCC and provide additional information for clinical decision-making.

Radiomics can extract abundant high-dimensional information from medical images and characterize the heterogeneity within tumors comprehensively and accurately. Tumors with different Ki-67 expression levels have been reported to exhibit significant heterogeneity in terms of cell proliferation and differentiation [24]. Therefore, by analyzing the radiomics features corresponding to tumors with different Ki-67 expression levels, the correlation between these characteristics and their potential biological significance can be explored. By transforming CT or MRI images into high-throughput quantitative data, radiomics features have been used to predict the Ki-67 index in various tumor types [17–20, 32, 33]. In this study, 13 optimal radiomics features were screened for their correlation with Ki-67 expression level in HNSCC, including nine wavelet transformed features, three first-order statistical features, and one filter transformed feature. The wavelet transformed features obtained by wavelet decomposition of the first order and texture features can extract heterogeneity information from the original images [34]. The wavelet features mainly include the gray level size zone matrix (GLSZM), gray level dependence matrix (GLDM), and first-order features. GLSZM is the number of linker voxels with the same gray intensity, while GLDM is the number of linker voxels within a specific distance dependent on the central voxel. The above two texture parameters are calculated values based on voxel alignment, which can characterize the irregularity of voxel alignment in the tumor space. Additionally, tumor heterogeneity may be related to local tumor cell number, proliferation, hypoxia, angiogenesis, and necrosis [35], and these factors are closely related to Ki-67 expression levels. This further suggests that medical image-based radiomics analysis can reflect tumor heterogeneity by describing the voxel arrangement in the tumor space.

In recent years, MRI has become an indispensable part of radiomics analysis due to its ultra-high soft-tissue resolution, absence of ionizing radiation, and multiparametric imaging capabilities. Previous MRI-based radiomics studies mainly focused on the evaluation of the staging [36], prognosis [37, 38], and treatment efficacy [39] of HNSCC. Ren et al. [36] reported an MRI-based radiomics signature using combined T2WI-FS and CE-T1WI images for the preoperative assessment of stage I-II and III-IV HNSCC with an AUC of 0.850 in the training cohort, which was higher than that of the radiomics signatures based on T2WI-FS images (AUC: 0.818) and CE-T1WI images (AUC: 0.828) alone, indicating that combined sequences can more comprehensively mine the heterogeneous features of the tumor. The advantage of multiparametric MRI was also confirmed in the study of Khanfari et al. [40]. Thus, we extracted the radiomics features from these two conventional MRI images to predict the Ki-67 expression level in HNSCC. The results showed that both sequences contributed to radiomics signature construction (seven features from T2WI-FS and six features from CE-T1WI). Then, based on the selected features, we used various ML classifiers to generate radiomics signatures, among which the SVM classifier showed the best performance in the validation cohorts and was selected as the optimal classifier. One possible explanation for this result is that the SVM algorithm usually seeks the best balance between complexity and learning ability, which can facilitate maximum generalizability in limited sample data [41]. In the present study, the SVM-based radiomics model using the combined sequences had higher AUC values (training cohort AUC: 0.884, validation cohorts average AUC: 0.851) than the models based on a single sequence. The results are comparable to a previous study that established a CT-based radiomics model to predict Ki-67 expression in HNSCC, with AUCs of 0.919 and 0.825 in the training and validation cohorts, respectively [22]. However, it should be noted that the soft-tissue resolution of CT images is poorer than that of MR images, and it may be challenging to precisely distinguish tumor boundaries in clinical practice by outlining ROIs. In addition, the high level of ionizing radiation generated by CT is a key concern for operators and patients.

This study also confirmed that clinical T stage and MR-reported LN status were significantly associated with the Ki-67 expression level in HNSCC. Significantly more HNSCC patients in the T3-T4 stage were present among those with a high Ki-67 index than among those with a low Ki-67 index, similar to a previous study [6]. This may be because tumors with high Ki-67 expression grow faster, are more aggressive, and are more likely to exhibit invasive growth and invade surrounding tissues. MR-reported LN status is another essential predictor. Liu et al. [42] indicated that the Ki-67 index correlated with the LN metastasis of HNSCC, and Gadbail et al. [43] found that the Ki-67 index was significantly higher in oral squamous cell carcinoma patients with LN metastasis. Our results were consistent with these findings. Nevertheless, the clinical model constructed based on the above two features only showed moderate performance, with AUCs of 0.737, 0.715, and 0.654 in the training, internal validation, and external validation cohorts, respectively. This is because clinical characteristics provide only visually observed anatomical data and cannot adequately reflect intra-tumoral heterogeneity. Therefore, we further integrated Rad-scores with clinical features to establish the fusion model. The incorporation of Rad-scores led to a significant increase in predictive efficiency and classification accuracy for the clinical model. However, there was no significant difference between the fusion model and the radiomics model in terms of AUCs, DCA, NRI, and IDI in the validation cohorts, which further confirmed the limitations of clinical features and highlighted the unique advantage of the radiomics signature in predicting Ki-67 expression levels.

Our study has some limitations. First, it is a retrospective study with unavoidable bias and a limited sample size. Further prospective studies and datasets with larger sample sizes from more centers are required to validate our prediction model. Second, we only adopted T2WI-FS and CE-T1WI images without diffusion-weighted images (DWI), because a significant proportion of patients lacked DWI. Given the potential value of DWI in radiomics analysis, subsequent studies should consider to incorporate DWI when available. Third, only HNSCC patients with a tumor maximal diameter beyond 5 mm were included in this study to obtain better tumor boundaries and sufficient pixel size for radiomics analysis. To broaden the model’s applicability, future research should consider including HNSCC patients with smaller tumor diameters. Fourth, due to the substantial disparities in prognosis and treatment response between nasopharyngeal carcinoma (NPC) and HNSCC at other sites, NPC patients were not included in this study. Thus, further parallel research on NPCs would be beneficial. Finally, manual segmentation is complex and time-consuming, thereby an automated, reliable, and reproducible segmentation method is required to develop in the future [44].

## Conclusions

In summary, we developed and validated an ML-based fusion model and corresponding nomogram that incorporated multiparametric MRI radiomics features and clinical factors to preoperatively predict the Ki-67 expression level in HNSCC patients. The risk calculated based on the nomogram helps to identify HNSCC patients with different risks of high Ki-67 expression, thereby identifying which patients have highly aggressive tumor and poor prognosis. Thus, this prediction model can provide important supplementary information to evaluate prognosis and guide treatment decisions.

### Supplementary Information

**Supplementary Material 1.**

## Data Availability

The datasets generated and/or analysed during the current study are not publicly available due [REASON WHY DATA ARE NOT PUBLIC] but are available from the corresponding author on reasonable request.

## Abbreviations

AUC: Area under the curve
CE-T1WI: Contrast-enhanced T1-weighted
CI: Confidence interval
DCA: Decision curve analysis
GLDM: Gray level dependence matrix
GLSZM: Gray level size zone matrix
HNSCC: Head and neck squamous cell carcinoma
ICC: Intra-class correlation coefficient
KNN: K-nearest neighbors
LASSO: Least absolute shrinkage and selection operator
LDA: Linear discriminant analysis
LR: Logistic regression
LN: Lymph node
ML: Machine learning
NB: Naive Bayes
OR: Odds ratio
Rad-score: Radiomics score
RF: Random forest
ROC: Receiver operating characteristic
ROI: Region of interest
SVM: Support vector machine
T2WI-FS: T2-weighted imaging fat suppression
VIF: Variance inflation factor
VOI: Volume of interest
XGBoost: EXtreme Gradient Boosting
