# Supplementary material for: Prediction of the Ki-67 expression level in head and neck squamous cell carcinoma with machine learning-based multiparametric MRI radiomics: a multicenter study
Source: BMC Cancer. 2024 Apr 5;24:418. doi: 10.1186/s12885-024-12026-x (PMC10996101; doi:10.1186/s12885-024-12026-x)

**Prediction of the Ki-67 expression level in head and neck squamous cell carcinoma with machine learning-based multiparametric MRI radiomics: a multicenter study**

**Supplementary Materials**

1. **Supplementary Methods**

**Appendix E1.** Inclusion and exclusion criteria for patients with head and neck squamous cell carcinoma

**Appendix E2.** Sample size estimation

**Appendix E3.** The classification criterion of MR-reported lymph node status

**Appendix E4.** Details of the extracted features

**Appendix E5.** Repeatability analysis

**Appendix E6.** Methods of Z-score normalization

**Appendix E7.** The three-step procedure of feature selection

**Appendix E8.** The rationales and considerations behind the choice of the seven machine learning (ML) classifiers

**Appendix E9.** The optimal hyperparameter combinations for each machine learning classifier

1. **Supplementary Tables**

**Table S1.** The sequences and parameters of different MR devices

**Table S2.** Comparison of the clinical characteristics and MRI features of HNSCC patients in the training, internal validation and external validation cohorts.

**Table S3.** Univariate and multivariate logistic regression analysis predicting the high Ki-67 expression index for HNSCC patients in the training cohort.

**Table S4.** The optimal radiomics feature subsets selected from the images of T2-weighted imaging fat suppression (T2WI-FS) and contrast-enhanced T1-weighted imaging (CE-T1WI).

1. **Supplementary Figures**

**Figure S1.** The results of radiomics feature selection. LASSO, least absolute shrinkage and selection operator; T2WI-FS, T2-weighted imaging fat suppression; CE-T1WI, contrast-enhanced T1-weighted imaging; MRI, magnetic resonance imaging.

**Figure S2. (A)** Feature coefficients corresponding to the value of parameter λ. Each curve represents the change trajectory of each independent variable. (**B)** The most valuable features were screened out by tuning λ using LASSO via minimum binomial deviation. As the parameter λ increases, the binominal deviance decreases gradually to the lowest point. The dotted vertical line represents the optimal log (λ) value. (**C)** The selected 13 radiomics features from a combination of T2-weighted and contrast-enhanced T1-weighted images with the most discriminative value.

**Figure S3.** The correlation heatmap of the selected radiomics features from a combination of T2-weighted imaging and contrast-enhanced T1-weighted imaging.

**Figure S4.** The receiver operating characteristic curves of radiomics signatures with seven machine learning classifiers in the (**A**) training, (**B**) internal validation, and (**C**) external validation cohorts. AUC, area under the curve; KNN, k-nearest neighbors; SVM, support vector machine; LR, logistic regression; RF, random forest; LDA, linear discriminant analysis; NB, naive Bayes; XGBoost, eXtreme Gradient Boosting.

**Figure S5.** The receiver operating characteristic curves and results of Delong tests of radiomics models using a single sequence and combined sequences of T2-weighted imaging (T2WI) and contrast-enhanced T1-weighted imaging (CE-T1WI) based on the support vector machine classifier in the (**A**) training, (**B**) internal validation, and (**C**) external validation cohorts. AUC, area under the curve.

**Figure S6.** Distribution of Rad-scores based on the SVM classifier between the Ki-67 low- and high-expression groups in the (**A**) training, (**B**) internal validation, and (**C**) external validation cohorts. Correlation analyses between the Ki-67 status, clinical features (clinical T stage and MR-reported LN), and radiomics features (Rx) in the (**D**) training, (**E**) internal validation, and (**F**) external validation cohorts. Rad-scores, radiomics scores; SVM, support vector machine; LN, lymph node.

**Figure S7.** Performance evaluation of different models for Ki-67 expression level prediction with net reclassification improvement (NRI) and integrated discrimination improvement (IDI). The NRI (A) and IDI (B) in the training cohort (left), internal validation cohort (middle) and external validation cohort (right) were shown inside the blocks, the responding significance were shown as the color of the blocks (blue for *P* ≥ 0.05 and red for *P* < 0.05).

**Appendix E1**

**Inclusion and exclusion criteria for patients with head and neck squamous cell carcinoma**

Inclusion criteria are as follows:

1. Biopsy-proven or surgically confirmed head and neck squamous cell carcinoma (HNSCC);
2. Postoperative immunohistochemical analysis of the Ki-67 expression level with accurate results;
3. Magnetic resonance imaging (MRI) examination of the head and neck within 2 weeks before surgery or puncture;
4. Complete clinical and pathological data.

Exclusion criteria are as follows:

1. HNSCC involving the nasopharynx, nasal cavity, paranasal sinuses, salivary glands, thyroid, or skin;
2. Tumor maximal diameter on axial images of ≤ 5 mm;
3. Receiving anti-tumor therapies before MRI examination;
4. Insufficient quality of MRI images, such as obvious motion artifact interference.

**Appendix E2**

**Sample size estimation**

For the training cohort, the assumptions made during the sample size calculation were a case-to-noncase ratio of 1:1, a desired AUC of 0.90, a confidence internal width of 0.15, and a two-sided α level of 0.05. Without considering the design effect of the dropout rate, the total sample size was 72, with 36 high Ki-67 expression cases and 36 low Ki-67 expression cases. Therefore, we believe that the sample size of our model in the training cohort (196, with 92 high Ki-67 expression cases and 104 low Ki-67 expression cases) meets the statistical test efficiency requirement.

For the validation cohorts, the split ratio of the training and internal validation cohort was 7:3. According to this ratio, the sample size needed in the validation cohorts was calculated to be 32, with 16 high Ki-67 expression cases and 16 low Ki-67 expression cases. In our study, 84 patients from center 1 (high Ki-67 expression: 40, low Ki-67 expression: 44) were included in the internal validation cohort, and 71 patients from center 2 were also enrolled as an external validation cohort, exceeding the minimum required sample sizes.

**Appendix E3**

**The classification criterion of MR-reported lymph node status**

MR-reported lymph node (LN) status recorded as positive required at least one of the following criteria: (1) short diameter of LNs of ≥ 10 mm on the largest cross-sectional image; (2) presence of central necrosis or circumferential enhancement at the edges; and (3) ≥ 3 LNs in the same high-risk area, with the short diameter of each LN being ≥ 8 mm[1].

**Appendix E4**

**Details of the extracted features**

The 1688 radiomics features used by Radcloud can be grouped into four groups: (1) 324 first-order features, which quantify the MR image’s voxel intensity distribution, including energy, entropy, mean, and median; (2) 14 shape features, which reflect the shape and size of the region, involving mesh surface, pixel surface, perimeter, and maximum diameter; (3) 525 texture features, which evaluate regional heterogeneity differences, including gray level co-occurrence matrix (GLCM), gray level dependence matrix (GLDM), gray level run length matrix (GLRLM), gray level size zone matrix (GLSZM), and neighborhood gray tone difference matrix (NGTDM); (4) 825 filter and wavelet features, which include the intensity and texture features derived from filter transformation and wavelet transformation of the original image, obtained by applying filters such exponential, gradient, logarithm, square, square root, lbp2D, and wavelet using eight frequency band combinations (low-high-low [LHL], low-high-high [LHH], high-low-low [HLL], low-low-high [LLH], high-low-high [HLH], high-high-high [HHH], high-high-low [HHL], and low-low-low [LLL]).

**Appendix E5**

**Repeatability analysis**

To ensure the reproducibility of the extracted features, one radiologist (reader A, with 7 years of experience) performed a second delineation of volumes of interest (VOIs) from 30% of randomly selected images (105 samples) 2 weeks later to evaluate the intra-observer repeatability. Then, another radiologist (reader B, with 16 years of experience) independently drew the VOIs from selected images to assess the inter-observer repeatability. The intra- and inter-observer repeatability were measured by the intraclass correlation coefficients (ICCs). Features with ICCs greater than 0.8 were considered to have good reproducibility and were included in subsequent analyses.

**Appendix E6**

**Methods of Z-score normalization**

The standardized formula is as follows:

$$f\left( x \right)=\frac{s(x-\mu_{x}）}{\delta_{x}}$$

where $x$ is the original intensity, $f\left( x \right)$ is the normalized intensity, $\mu$ and $\delta$ are the mean and variance, respectively, and $s$ is an optional scaling factor (set to 1 by default).

**Appendix E7**

**The three-step procedure of feature selection**

First, the variance threshold method was performed to initially select features with values of variance higher than 0.8. Second, the SelectKBest method was used to select features with *P* values of less than 0.05, which was based on univariate analysis with Pearson’s correlation test for continuous features and the chi-square test for categorical features. Third, the least absolute shrinkage and selection operator (LASSO) regression with 10-fold cross-validation was used to select the optimal features according to the best parameter (alpha).

**Appendix E8**

**The rationales and considerations behind the choice of the seven machine learning (ML) classifiers**

The k-nearest neighbors (KNN) algorithm classifies a data point based on how its features are similar to those of points in a dataset, considering the ‘k’ closest points (‘neighbors’) to it[2]. It was chosen for its utility in classification tasks where simplicity and interpretability are crucial. The support vector machine (SVM) algorithm can be used for linear or non-linear classification, which aims to find the optimal segmentation hyperplane to make the points closer to the hyperplane have a larger distance[3]. The effectiveness in high-dimensional spaces made it an ideal choice for handling the complex feature sets typical of radiomics data. Logistic regression (LR) is a commonly used binary linear classifier, which uses the sigmoid function for non-linear transformation and the log maximum likelihood estimation function for learning the posterior probability of a single sample[4]. LR was included for its proficiency in providing probabilistic outputs and for ease of interpretation in a clinical context. The random forest (RF) algorithm assesses the importance of individual features in prediction by evaluating the reduction of impurity in trees within the forest attributable to each feature, and automatically calculates the standardized score of each feature after training[5]. RF was selected for its ability to use ensemble learning from multiple decision trees to improve prediction accuracy. The linear discriminant analysis (LDA) algorithm projects the data on the low dimension, and its purpose is to select the projection direction with the best classification performance[6]. LDA was chosen for its strength in dimensionality reduction and classification tasks, particularly in datasets where linear combinations of features are meaningful. The naive Bayes (NB) algorithm is based on applying Bayes’ theorem with the “naive” assumption of conditional independence between every pair of features[7]. It’s particularly effective for categorical data and is commonly used for classification tasks. The eXtreme Gradient Boosting (XGBoost) algorithm operates by constructing a sequence of decision trees, where each subsequent tree corrects the errors made by the previous ones[8]. It was chosen for its high efficiency, flexibility, and capability to handle a variety of data types and tasks. Each classifier was chosen for its unique approach to learning and pattern recognition, ensuring that the model captured various aspects of the data. More importantly, these are commonly used classifiers in previous ML related studies[9-11]. The inclusion of different ML classifiers allows us to compare and construct the optimal prediction model for our specific application.

**Appendix E9**

**The optimal hyperparameter combinationsfor each machine learning classifier**

The optimal hyperparameter combinations for the seven classifiers are as follows:

1. KNN: n_neighbors=5;
2. SVM: C=0.6, kernel=‘rbf’;
3. LR: penalty=‘l2’, C=0.5, solver=‘lbfgs’, class_weight =‘balanced’;
4. RF: n_estimators = 10, max_depth=3;
5. LDA: Solver=‘svd’;
6. NB: priors=None, var_smoothing= 1e-09;
7. XGBoost: n_estimators=50, max_depth= 6.

**References:**

[1] van den Brekel MW, Stel HV, Castelijns JA, Nauta JJ, van der Waal I, Valk J, Meyer CJ, Snow GB: Cervical lymph node metastasis: assessment of radiologic criteria. Radiology 1990, 177(2):379-384.

[2] Yosipof A, Senderowitz H: k-Nearest neighbors optimization-based outlier removal. Journal of computational chemistry 2015, 36(8):493-506.

[3] Luts J, Ojeda F, Van de Plas R, De Moor B, Van Huffel S, Suykens JA: A tutorial on support vector machine-based methods for classification problems in chemometrics. Analytica chimica acta 2010, 665(2):129-145.

[4] LaValley MP: Logistic regression. Circulation 2008, 117(18):2395-2399.

[5] Rigatti SJ: Random Forest. Journal of insurance medicine (New York, NY) 2017, 47(1):31-39.

[6] Keysers D, Ney H: Linear Discriminant Analysis and Discriminative Log-linear Modeling. In: International Conference on Pattern Recognition: 2004; 2004.

[7] Ahmed MS, Shahjaman M, Rana MM, Mollah MNH: Robustification of Naïve Bayes Classifier and Its Application for Microarray Gene Expression Data Analysis. BioMed research international 2017, 2017:3020627.

[8] Ma B, Meng F, Yan G, Yan H, Chai B, Song F: Diagnostic classification of cancers using extreme gradient boosting algorithm and multi-omics data. Computers in biology and medicine 2020, 121:103761.

[9] Mukherjee S, Patra A, Khasawneh H, Korfiatis P, Rajamohan N, Suman G, Majumder S, Panda A, Johnson MP, Larson NB et al: Radiomics-based Machine-learning Models Can Detect Pancreatic Cancer on Prediagnostic Computed Tomography Scans at a Substantial Lead Time Before Clinical Diagnosis. Gastroenterology 2022, 163(5):1435-1446.e1433.

[10] Chen B, Mao Y, Li J, Zhao Z, Chen Q, Yu Y, Yang Y, Dong Y, Lin G, Yao J et al: Predicting very early recurrence in intrahepatic cholangiocarcinoma after curative hepatectomy using machine learning radiomics based on CECT: A multi-institutional study. Computers in biology and medicine 2023, 167:107612.

[11] Chen Y, Xia Y, Tolat PP, Long L, Jiang Z, Huang Z, Tang Q: Comparison of Conventional Gadoxetate Disodium-Enhanced MRI Features and Radiomics Signatures With Machine Learning for Diagnosing Microvascular Invasion. AJR American journal of roentgenology 2021, 216(6):1510-1520.

**Table S1.** The sequences and parameters of different MR devices

|  | Sequence | TR (ms) | TE (ms) | Matrix | Layer thickness (mm) | NSA |
| --- | --- | --- | --- | --- | --- | --- |
| Center 1 | T2WI-FS | 3000 | 90 | 288×185 | 4 | 4 |
|  | CE-T1WI | 480 | 18 | 260×153 | 4 | 2 |
| Center 2 | T2WI-FS | 3000 | 65 | 288×224 | 4 | 2 |
|  | CE-T1WI | 400 | 10 | 256×192 | 4 | 2 |

**Abbreviations:** CE-T1WI, contrast-enhanced T1-weighted imaging; T2WI-FS, T2-weighted imaging fat suppression; TR, time of repetition; TE, time of echo; NSA, number of signal averages.

**Table S2.** Comparison of the clinical characteristics and MRI features of HNSCC patients in the training, internal validation and external validation cohorts.

| **Characteristic** | **Training cohort**  **(n =196)** | **Internal validation cohort**  **(n = 84)** | **External validation cohort**  **(n = 71)** | ***P* value** |
| --- | --- | --- | --- | --- |
| Age, years |  |  |  | 0.835 |
| < 60 | 86 (43.9) | 37 (44.0) | 34 (47.9) |  |
| ≥ 60 | 110 (56.1) | 47 (56.0) | 37 (52.1) |  |
| Sex |  |  |  | 0.988 |
| Male | 153 (78.1) | 66 (78.6) | 56 (78.9) |  |
| Female | 43 (21.9) | 18 (21.4) | 15 (21.1) |  |
| Smoking history |  |  |  | 0.780 |
| No | 115 (58.7) | 51 (60.7) | 45 (63.4) |  |
| Yes | 81 (41.3) | 33 (39.3) | 26 (36.6) |  |
| Tumor location |  |  |  | 0.891 |
| Oral cavity | 75 (38.3) | 33 (39.3) | 28 (39.4) |  |
| Oropharynx | 28 (14.3) | 11 (13.1) | 9 (12.7) |  |
| Larynx | 63 (32.1) | 32 (38.1) | 25 (35.2) |  |
| Hypopharynx | 30 (15.3) | 8 (9.5) | 9 (12.7) |  |
| Clinical T stage |  |  |  | 0.752 |
| T1-T2 | 87 (44.4) | 41 (48.8) | 34 (47.9) |  |
| T3-T4 | 109 (55.6) | 43 (51.2) | 37 (52.1) |  |
| MR-reported LN status |  |  |  | 0.954 |
| Negative | 120 (61.2) | 51 (60.7) | 42 (59.2) |  |
| Positive | 76 (38.8) | 33 (39.3) | 29 (40.8) |  |

**Abbreviations:** HNSCC, head and neck squamous cell carcinoma; LN, lymph node; MRI, magnetic resonance imaging.

**Table S3.** Univariate and multivariate logistic regression analysis predicting the high Ki-67 expression index for HNSCC patients in the training cohort.

**Abbreviations:** CI, confidence interval; HNSCC, head and neck squamous cell carcinoma; LN, lymph node; OR, odds ratio.

| **Variable** | **OR Comparison** | **Univariate analysis** | | **Multivariate analysis** | |
| --- | --- | --- | --- | --- | --- |
|  |  | **OR (95% CI)** | ***P* value** | **OR (95% CI)** | ***P* value***** |
| Age (years) | ≥ 60 *vs.* < 60 | 1.197 (0.554–2.582) | 0.647 |  |  |
| Sex | Male *vs.* Female | 0.619 (0.244-1.570) | 0.312 |  |  |
| Smoking history | Yes *vs.* No | 0.889  (0.410-1.929) | 0.766 |  |  |
| Tumor location | Oropharynx *vs.* Oral cavity | 1.917 (0.575-6.383) | 0.289 |  |  |
|  | Larynx *vs.* Oral cavity | 1.009  (0.404-2.521) | 0.985 |  |  |
|  | Hypopharynx *vs.* Oral cavity | 1.278  (0.401-4.068) | 0.678 |  |  |
| Clinical T stage | T3-T4 *vs.* T1-T2 | 0.644 (0.327-1.271) | 0.644 | 3.715  (1.580-8.737) | **0.003** |
| MR-reported LN status | Positive *vs.* Negative | 0.477 (0.126-1.808) | 0.477 | 2.836  (1.195-6.729) | **0.018** |

**Table S4.** The optimal radiomics feature subsets selected from the images of T2-weighted imaging fat suppression (T2WI-FS) and contrast-enhanced T1-weighted imaging (CE-T1WI).

| **Sequence** | **T2WI-FS** | **CE-T1WI** | **T2WI-FS+CE-T1WI** |
| --- | --- | --- | --- |
| NF | 8 | 8 | 13 |
| 1 | T2W_original_shape_LeastAxisLength | CE-T1W_original_shape_LeastAxisLength | T2W_original_firstorder_InterquartileRange |
| 2 | T2W_original_shape_Maximum2DDiameterSlice | CE-T1W_wavelet_LLH_glszm_GrayLevelNonUniformity | CE-T1W_wavelet_LLH_glszm_GrayLevelNonUniformity |
| 3 | T2W_original_glszm_SizeZoneNonUniformity | CE-T1W_wavelet_LHL_glszm_GrayLevelNonUniformity | T2W_wavelet_LLH_firstorder_InterquartileRange |
| 4 | T2W_wavelet_LLH_firstorder_InterquartileRan ge | CE-T1W_wavelet_HLH_glszm_ZoneEntropy | T2W_wavelet_LLH_firstorder_Variance |
| 5 | T2W_wavelet_LLH_firstorder_Variance | CE-T1W_wavelet_LHL_glszm_SizeZoneNonUniformity | T2W_wavelet_LHH_glszm_HighGrayLevelZoneEmphasis |
| 6 | T2W_wavelet_LLH_firstorder_Kurtosis | CE-T1W_wavelet_HLL_glszm_SizeZoneNonUniformity | CE-T1W_wavelet_HLH_glszm_ZoneEntropy |
| 7 | T2W_lbp_3D_m1_firstorder_10Percentile | CE-T1W_wavelet_LLL_firstorder_Minimum | CE-T1W_wavelet_LLL_gldm_LargeDependenceHighGrayLevelEmphasis |
| 8 | T2W_gradient_firstorder_90Percentile | CE-T1W_wavelet_HLL_firstorder_Energy | CE-T1W_wavelet_HLL_firstorder_Energy |
| 9 |  |  | T2W_wavelet_LHL_glszm_SmallAreaHighGrayLevelEmphasis |
| 10 |  |  | CE-T1W_wavelet_HHH_glszm_HighGrayLevelZoneEmphasis |
| 11 |  |  | T2W_lbp_3D_m1_firstorder_90Percentile |
| 12 |  |  | T2W_lbp_3D_m1_firstorder_10Percentile |
| 13 |  |  | CE-T1W_exponential_gldm_DependenceVariance |

**Figure S1.** The results of radiomics feature selection. LASSO, least absolute shrinkage and selection operator; T2WI-FS, T2-weighted imaging fat suppression; CE-T1WI, contrast-enhanced T1-weighted imaging; MRI, magnetic resonance imaging.


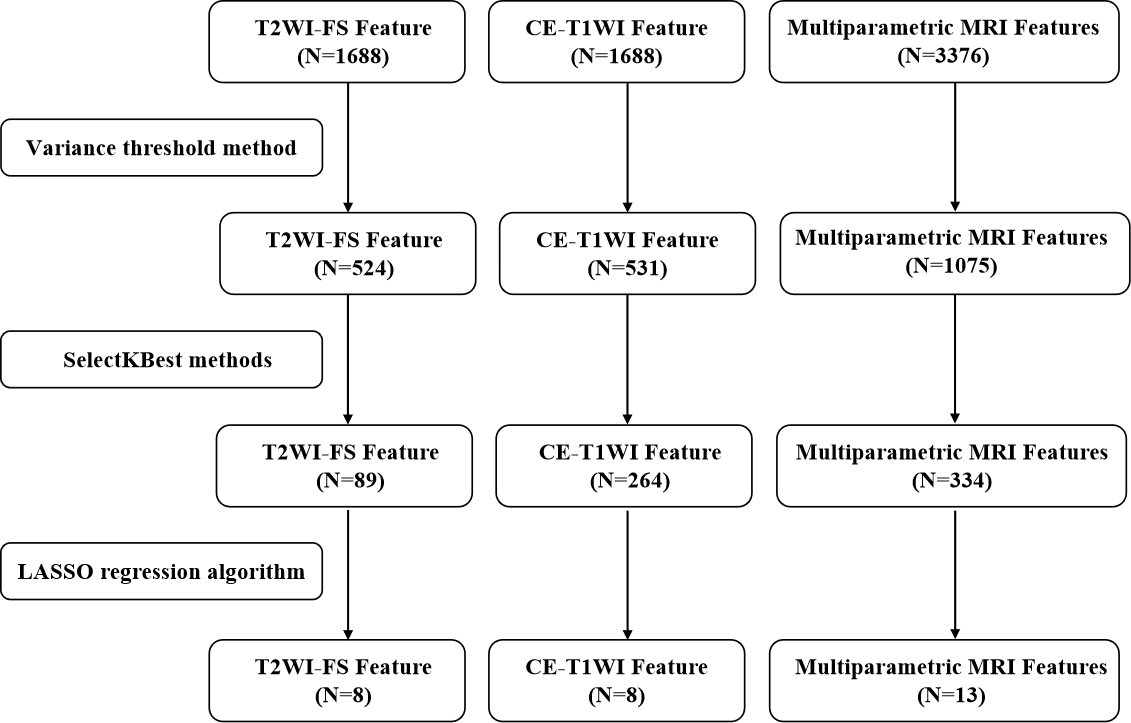


**Figure S2. (A)** Feature coefficients corresponding to the value of parameter λ. Each curve represents the change trajectory of each independent variable. (**B)** The most valuable features were screened out by tuning λ using LASSO via minimum binomial deviation. As the parameter λ increases, the binominal deviance decreases gradually to the lowest point. The dotted vertical line represents the optimal log (λ) value. (**C)** The selected 13 radiomics features from a combination of T2-weighted and contrast-enhanced T1-weighted images with the most discriminative value.


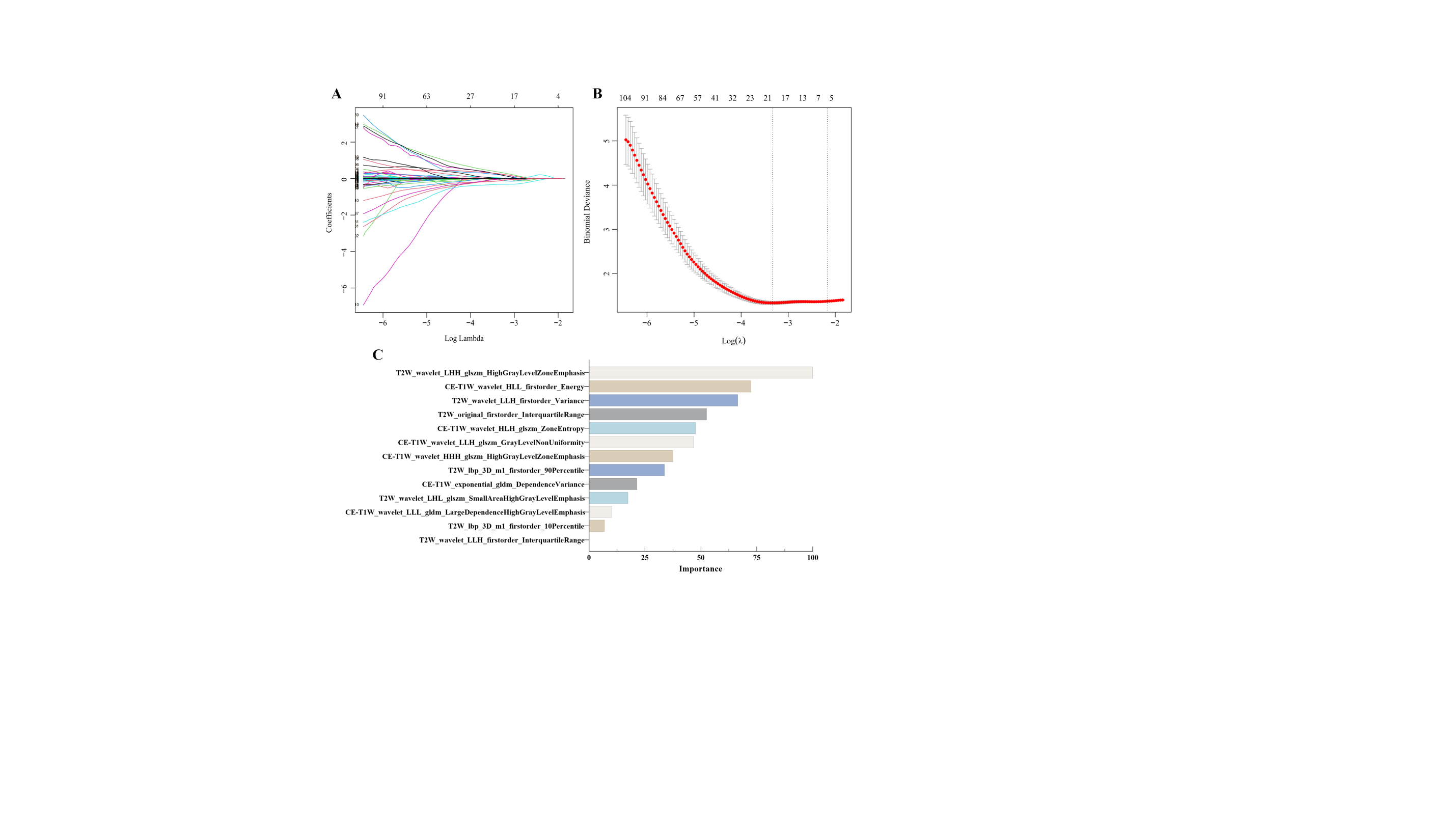


**Figure S3.** The correlation heatmap of the selected radiomics features from a combination of T2-weighted imaging and contrast-enhanced T1-weighted imaging.

**
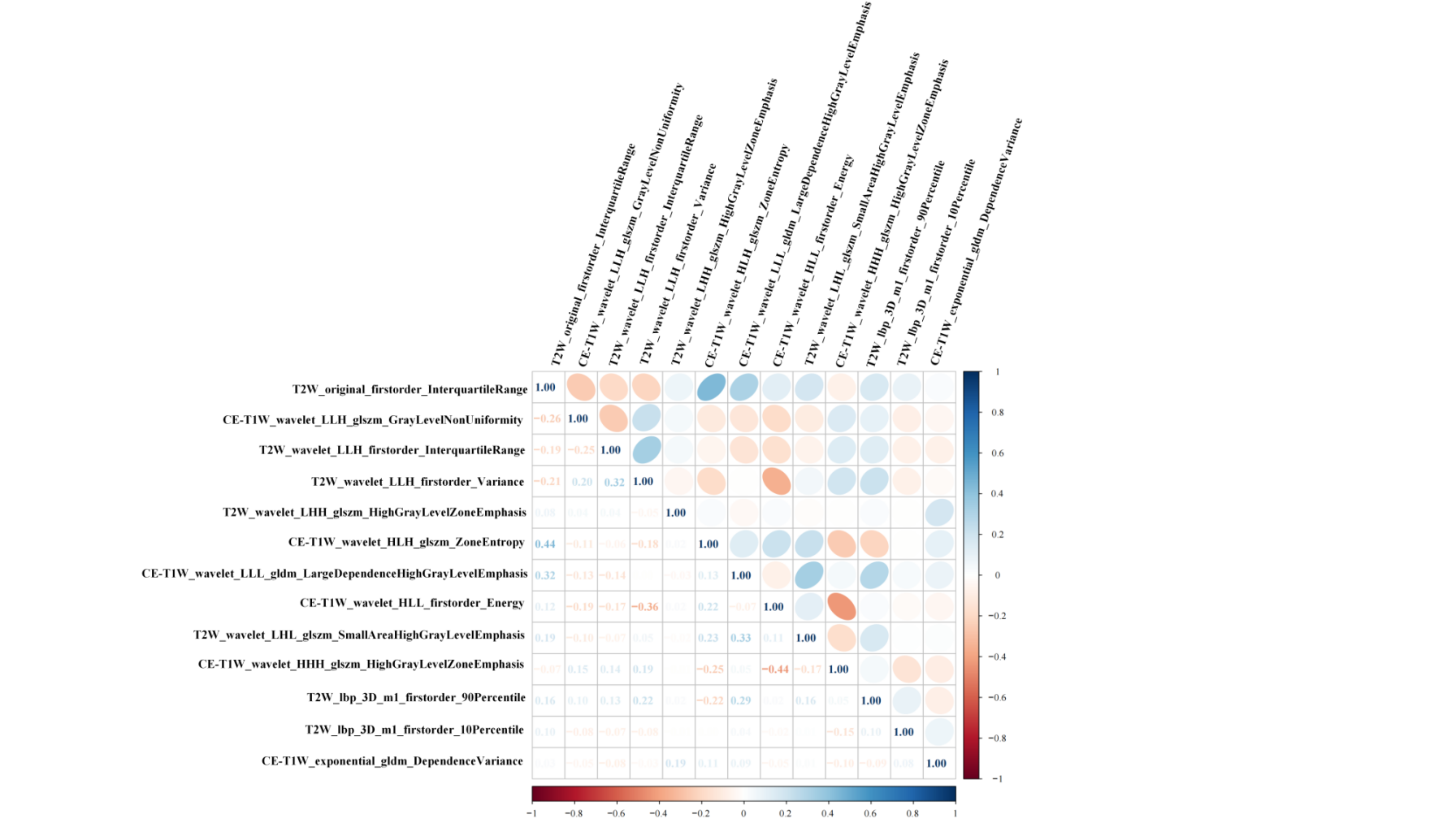
**

**Figure S4.** The receiver operating characteristic curves of radiomics signatures with seven machine learning classifiers in the (**A**) training, (**B**) internal validation, and (**C**) external validation cohorts. AUC, area under the curve; KNN, k-nearest neighbors; SVM, support vector machine; LR, logistic regression; RF, random forest; LDA, linear discriminant analysis; NB, naive Bayes; XGBoost, eXtreme Gradient Boosting.


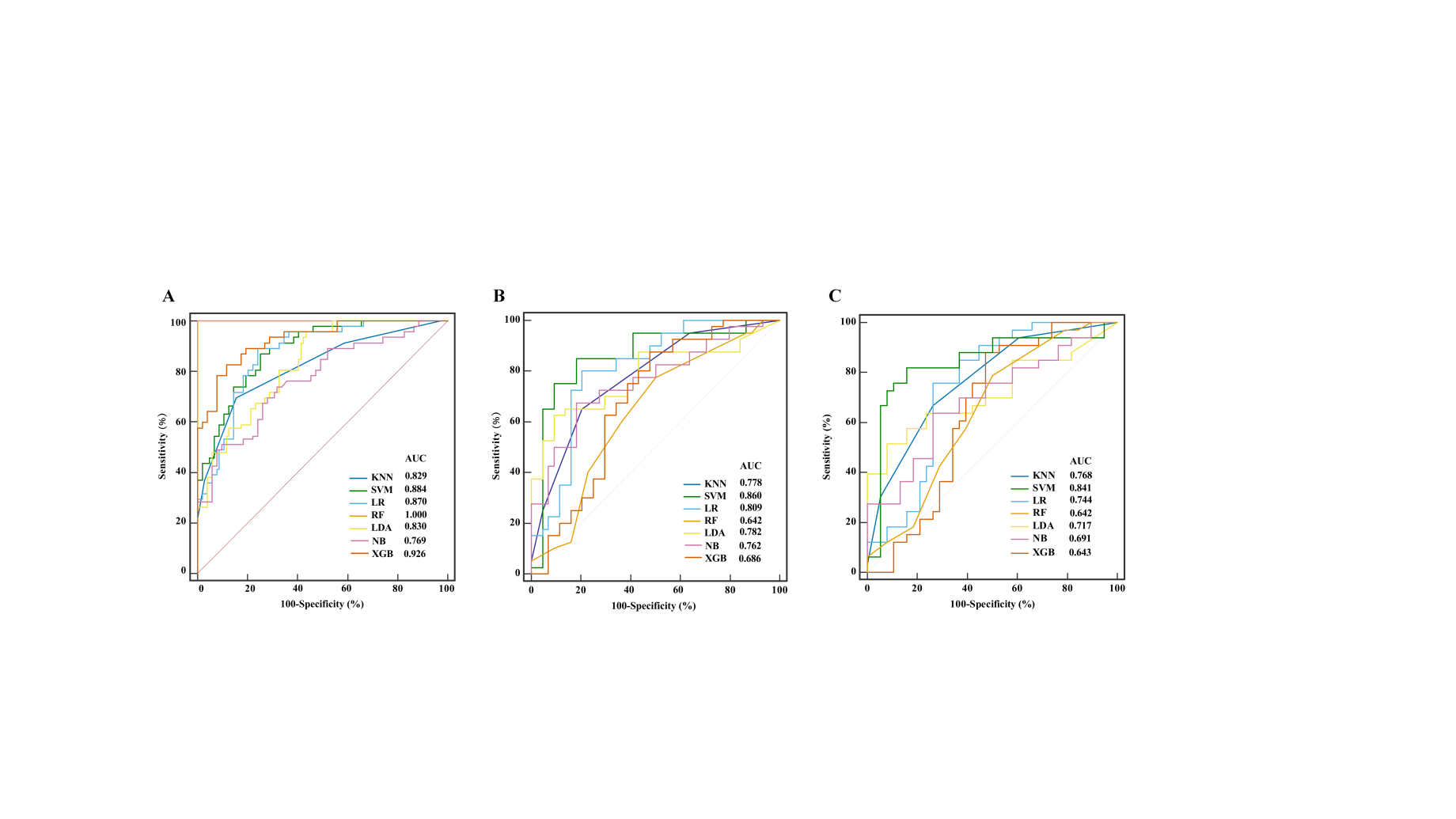


**Figure S5.** The receiver operating characteristic curves and results of Delong tests of radiomics models using a single sequence and combined sequences of T2-weighted imaging (T2WI) and contrast-enhanced T1-weighted imaging (CE-T1WI) based on the support vector machine classifier in the (**A**) training, (**B**) internal validation, and (**C**) external validation cohorts. AUC, area under the curve.


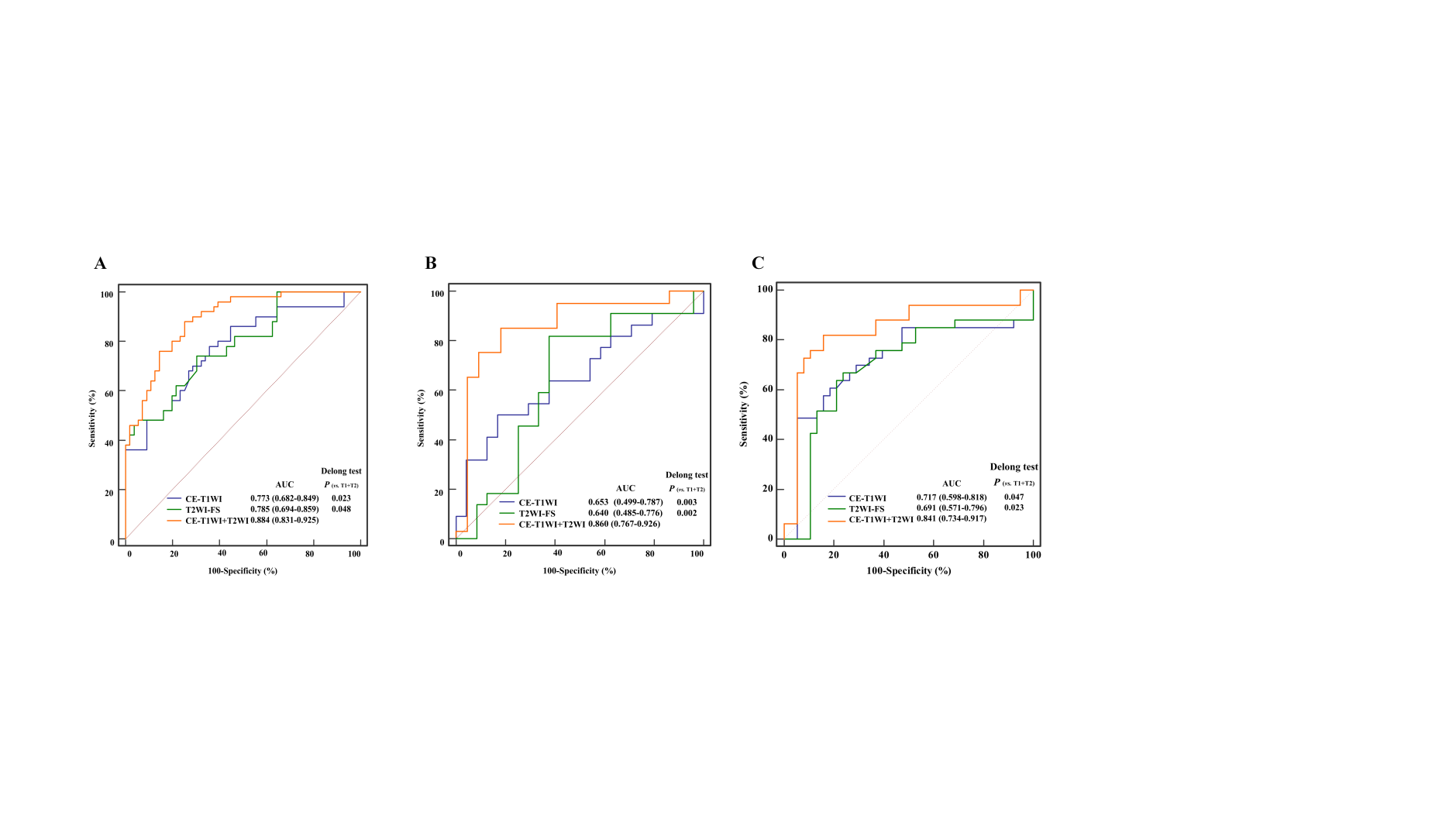


**Figure S6.** Distribution of Rad-scores based on the SVM classifier between the Ki-67 low- and high-expression groups in the (**A**) training, (**B**) internal validation, and (**C**) external validation cohorts. Correlation analyses between the Ki-67 status, clinical features (clinical T stage and MR-reported LN), and radiomics features (Rx) in the (**D**) training, (**E**) internal validation, and (**F**) external validation cohorts. Rad-scores, radiomics scores; SVM, support vector machine; LN, lymph node.


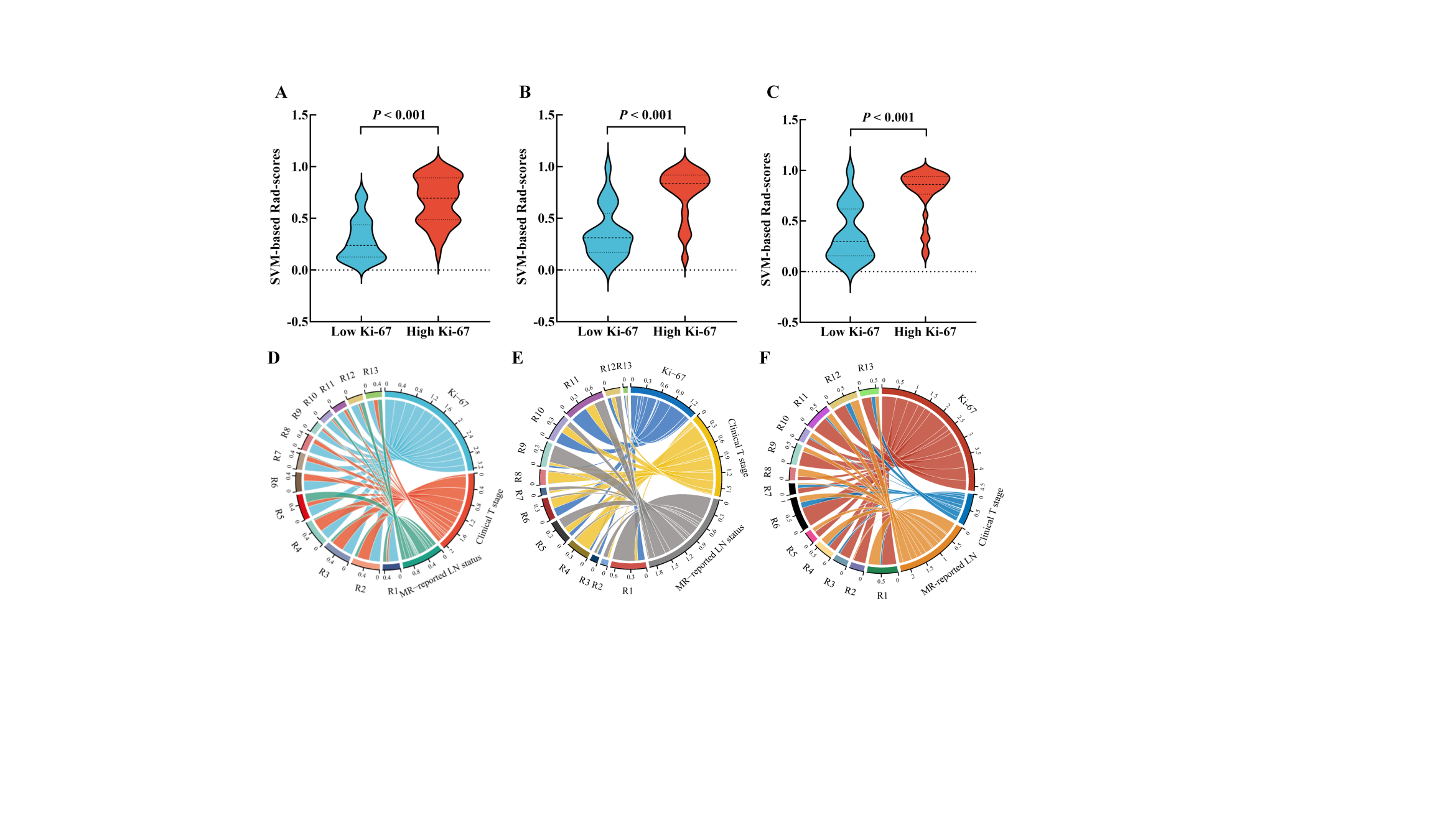


**Figure S7.** Performance evaluation of different models for Ki-67 expression level prediction with net reclassification improvement (NRI) and integrated discrimination improvement (IDI). The NRI **(A)** and IDI **(B)** in the training cohort (left), internal validation cohort (middle) and external validation cohort (right) were shown inside the blocks, the responding significance were shown as the color of the blocks (blue for *P* ≥ 0.05 and red for *P* < 0.05).


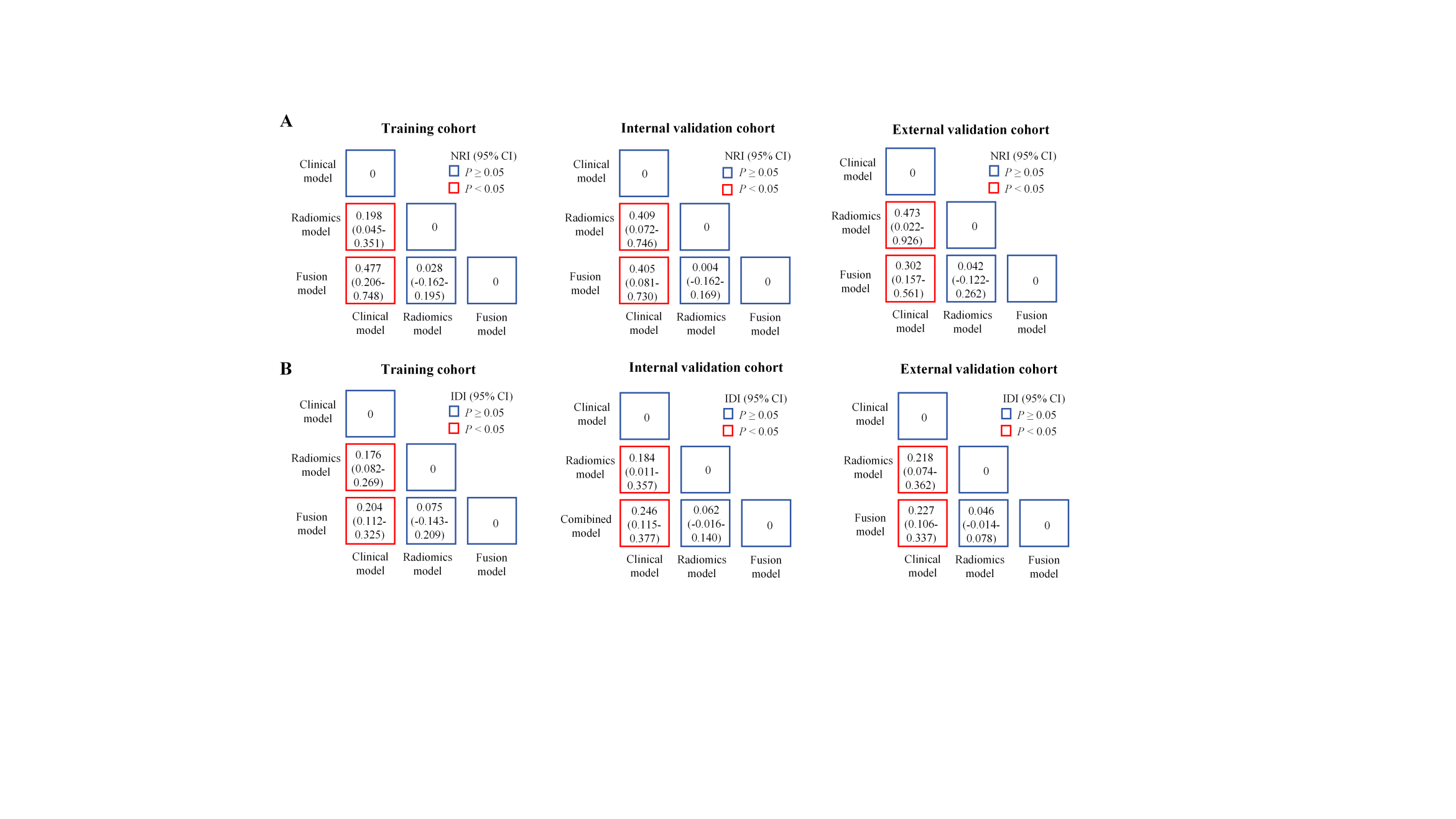

Supplement: Supplementary file 1 — Supplementary Material 1. [file 12885_2024_12026_MOESM1_ESM.docx]
